# Geographic, Occupational, and Sociodemographic Variations in Uptake of COVID-19 Booster Doses Among Fully Vaccinated US Adults, December 1, 2021, to January 10, 2022

**DOI:** 10.1001/jamanetworkopen.2022.27680

**Published:** 2022-08-19

**Authors:** Israel T. Agaku, Caleb Adeoye, Theodore G. Long

**Affiliations:** 1 NYC Test and Trace Corps, NYC Health + Hospitals, New York, New York

## Abstract

**Importance:**

COVID-19 booster vaccine can strengthen waning immunity and widen the range of immunity against new variants.

**Objective:**

To describe geographic, occupational, and sociodemographic variations in uptake of COVID-19 booster doses among fully vaccinated US adults.

**Design, Setting, and Participants:**

This cross-sectional survey study used data from the Household Pulse Survey conducted from December 1, 2021, to January 10, 2022. Household Pulse Survey is an online, probability-based survey conducted by the US Census Bureau and is designed to yield estimates nationally, by state, and across selected metropolitan areas.

**Main Outcomes and Measures:**

Receipt of a booster dose was defined as taking 2 or more doses of COVID-19 vaccines with the first one being the Johnson and Johnson (Janssen) vaccine, or taking 3 or more doses of any of the other COVID-19 vaccines. Weighted prevalence estimates (percentages) were computed overall and among subgroups. Adjusted prevalence ratios (APRs) were calculated in a multivariable Poisson regression model to explore correlates of receiving a booster dose among those fully vaccinated.

**Results:**

A total of 135 821 adults completed the survey. Overall, 51.0% were female and 41.5% were aged 18 to 44 years (mean [SD] age, 48.07 [17.18] years). Of fully vaccinated adults, the percentage who reported being boosted was 48.5% (state-specific range, from 39.1% in Mississippi to 66.5% in Vermont). Nationally, the proportion of boosted adults was highest among non-Hispanic Asian individuals (54.1%); those aged 65 years or older (71.4%); those with a doctoral, professional, or master’s degree (68.1%); those who were married with no children in the household (61.2%); those with annual household income of $200 000 or higher (69.3%); those enrolled in Medicare (70.9%); and those working in hospitals (60.5%) or in deathcare facilities (eg, funeral homes; 60.5%). Conversely, only one-third of those who ever received a diagnosis of COVID-19, were enrolled in Medicaid, working in pharmacies, with less than a high school education, and aged 18 to 24 years old were boosted. Multivariable analysis of pooled national data revealed that compared with those who did not work outside their home, the likelihood of being boosted was higher among adults working in hospitals (APR, 1.23; 95% CI, 1.17-1.30), ambulatory health care centers (APR, 1.16; 95% CI, 1.09-1.24), and social service settings (APR, 1.08; 95% CI, 1.01-1.15), whereas lower likelihood was seen among those working in food or beverage stores (APR, 0.85; 95% CI, 0.74-0.96) and the agriculture, forestry, fishing, or hunting industries (APR, 0.83; 95% CI, 0.72-0.97).

**Conclusions and Relevance:**

These findings suggest continuing disparities in receipt of booster vaccine doses among US adults. Targeted efforts at populations with low uptake may be needed to improve booster vaccine coverage in the US.

## Key Points

**Question:**

How has uptake of COVID-19 booster doses among fully vaccinated US adults varied by geographic location, occupation, and sociodemographic characteristics?

**Findings:**

In this cross-sectional survey study of 135 821 US adults aged 18 years or older who participated in the Household Pulse Survey from December 1, 2021, to January 10, 2022, less than half (48.5%) of individuals who had been fully vaccinated nationwide had received a booster dose. Marked variations were seen across geographic locations, occupation types, and other sociodemographic characteristics; boosted individuals were more likely than nonboosted individuals to be male, Asian, more educated and older, and to live in the Northeast and earn a high income, and work in hospitals.

**Meaning:**

These findings suggest that targeted efforts to increase booster vaccine coverage among subgroups with low uptake may benefit public health.

## Teaser

This survey study examines geographic, occupational, and sociodemographic variations in uptake of COVID-19 booster doses among fully vaccinated US adults.

## Introduction

COVID-19 vaccines are a crucial preventive measure against the pandemic.^1^ Between December 2020 and February 2021, the Food and Drug Administration issued an Emergency Use Authorization for 3 vaccines with high efficacy—Ad26.COV2.S (Johnson & Johnson–Janssen), mRNA-1273 (Moderna), and BNT162b2 (Pfizer-BioNTech)—the latter 2 of which have now received full Food and Drug Administration approval.^2,3,4^ More than 553 million doses of COVID-19 vaccines have been administered in the US, covering an estimated 65.2% of the US population.^5^

Early on, there was a recognition that certain special populations, such as older adults or immunocompromised patients, needed additional COVID-19 vaccine doses after completion of an initial COVID-19 vaccination series because of their lower immune response.^6,7^ The indicated population for booster doses was subsequently broadened on the basis of evidence of reduction in protection offered by the vaccines.^8,9,10,11^ Immunologic and epidemiologic studies performed on potency of COVID-19 vaccines showed reduction in antibody levels activated by the vaccines and increased incidence of breakthrough cases among fully vaccinated individuals.^1,12^ In addition, new variants emerged with the potential to evade vaccine immunity.^11,13^ Increased transmission of the Delta variant beginning in the spring of 2021 led to an increase in numbers of breakthrough COVID-19 infections.^1^ According to US Centers for Disease Control and Prevention (CDC) recommendations, all individuals aged 12 years or older should receive a COVID-19 mRNA vaccine booster dose 5 months after completion of an initial vaccination series.^9^ The administration of booster vaccines strengthens waning immunity and widens the range of immunity against new variants detected.^14^

Although studies^11,14,15,16^ have been performed to evaluate the need for and attitudes toward booster doses, to the best of our knowledge, no representative study has been conducted assessing the prevalence of booster dose uptake and factors associated with uptake. Understanding geographic, occupational, and sociodemographic differences in receipt of booster doses among the fully vaccinated population can help inform targeted efforts to increase coverage. The aim of this survey study was to explore prevalence and correlates of self-reported receipt of a booster vaccine dose among the fully vaccinated population. To explore place-based differences in booster vaccine uptake, prevalence estimates were computed and compared at the level of the major metropolitan statistical areas (MSAs), all 50 US states, and Washington, DC, as well as the US overall.

## Methods

### Data Source

Data were obtained from the Household Pulse Survey (HPS), an ongoing, biweekly, national and state-specific, COVID-19–related, cross-sectional survey of US adults aged 18 years or older.^17^ The HPS uses the US Census Bureau’s Master Address File as the source of sampled housing units. The sample design was a systematic sample of all eligible housing units, with adjustments applied to the sampling intervals to select a large enough sample to create state-level estimates and estimates for the top 15 MSAs. In terms of fielding the survey, the US Census Bureau conducts this information collection online using Qualtrics as the data collection platform. All survey initiations for the HPS are distributed to sampled participants via email and SMS, and data collection occurs entirely on the web. The data collection platform is optimized for use on a mobile device, so it may be used via any type of internet access. For this study, we analyzed pooled data from 2 survey cycles spanning the period December 1, 2021, to January 10, 2022. This was a unique period that marked the shift from Delta to Omicron as the dominant variant of COVID-19 diagnoses in the US. Between December 13, 2021, and January 10, 2022, the share of SARS-CoV-2 sequences that were the Omicron variant increased dramatically from 3.74% to 93.07%.^5^ Ethical review and informed consent were not sought because the secondary, publicly available data set was deidentified and lacked private information, in accordance with 45 CFR §46. This study follows the Strengthening the Reporting of Observational Studies in Epidemiology (STROBE) reporting guideline for observational studies and complies with ethical standards set by the American Association for Public Opinion Research (AAPOR) reporting guideline.^18,19^

### Measures

#### Receipt of a Full and Booster COVID-19 Vaccine Dose and COVID-19 Diagnosis

The survey asked participants whether they had ever “received a COVID-19 vaccine” (yes or no); “how many dose(s) of a COVID-19 vaccine” they had received (1, 2, 3, or 4 or more vaccinations) and the brand of first vaccination (“Pfizer-BioNtech”, “Moderna”, “Johnson and Johnson (Janssen)”, “One of the brands that requires two initial shots, but not sure which brand”, “None of these brands”, or “Don’t know”). These variables were used to create indicators for receipt of a full dose (ie, completing the initial vaccination schedule) and a booster dose. Participants were said to have received a full COVID-19 vaccine dose (ie, be fully vaccinated) if they reported taking at least 1 dose of the Johnson and Johnson (Janssen) COVID-19 vaccine or at least 2 doses of any other COVID-19 vaccines. Receipt of a booster dose was defined as taking at least 2 doses of COVID-19 vaccines with the first one being the Johnson and Johnson (Janssen) vaccine, which is only a 1-dose regimen, or taking at least 3 doses of any of the other vaccines that require an initial 2-dose regimen. Responses of “Don’t know” were set to missing to reduce misclassification bias. Self-reported diagnosis of COVID-19 was defined as a response of yes to the question, “Has a doctor or other healthcare provider ever told you that you have COVID-19?”

#### Sociodemographic Factors

We were interested in how receipt of a booster dose varied by occupational setting, especially among those working in jobs with frequent contact with individuals of unknown COVID-19 status. The survey asked, “In the last 7 days, which best describes the primary location/setting where you worked or volunteered outside your home?” A variety of settings was assessed (eg, hospital, K-12 school, or correctional facility) and only 1 response could be selected. Participants could also select if they did not work or volunteer “outside [their] home.” Other sociodemographic factors asked about in the survey included marital status, number of children in household, US Census region, race and ethnicity, gender, age, education, and type of housing (eg, multiunit dwelling). Race and ethnicity were assessed in this study to determine whether disparities existed in booster dose coverage.

### Statistical Analysis

Data (percentages) were weighted to yield representative estimates. The weights were provided in the online data set to account for the complex survey sampling. The final HPS weights are designed to produce biweekly estimates for the total persons aged 18 years or older living within housing units. These weights were created by adjusting the household-level sampling base weights by various factors to account for nonresponse, adults per household, and coverage.

The sampling base weights for each incoming sample in each of the sample areas are calculated as the total eligible housing units in the sampling frame divided by the number of eligible housing units selected for interviews each week. Therefore, the base weights for all sampled housing units sum to the total number of housing units for which contact information is known. Prevalence estimates were computed nationally, by state, and by the major MSAs. We expressed COVID-19 booster dose coverage both as prevalence in the overall population (numerator, received a booster dose; denominator, all adults including those vaccinated and unvaccinated), as well as a proportion among the fully vaccinated (numerator, received a booster dose; denominator, all fully vaccinated adults). Prevalence estimates with relative SEs 40% or higher were deemed imprecise and suppressed. Adjusted prevalence ratios (APRs) were calculated in an exploratory Poisson regression model to assess factors associated with receipt of a booster dose among those fully vaccinated. Prevalence ratios were calculated in lieu of odds ratios as they are more interpretable, consistent, and conservative, especially when the outcome is common,^20,21^ as is the case in our study. Independent variables assessed were household structure (marital status and number of children), US Census region, race and ethnicity, gender, age, education, type of housing, occupational sector, and ever COVID-19 diagnosis status. All analyses were performed in Stata statistical software version 14 (StataCorp). All statistical tests were 2-tailed, and statistical significance was set at *P* < .05.

## Results

### Descriptive Statistics

For our analyses, the pooled sample size was 135 821 participants. Baseline characteristics of study participants are presented in Table 1. Overall, 51.0% were female and 41.5% were aged 18 to 44 years (mean [SD] age, 48.07 [17.18] years). Age distributions were as follows: 18 to 24 years (7.9%), 25 to 44 years (33.6%), 45 to 64 years (34.8%), and 65 years and older (23.7%). The percentage of the US adult population that was fully vaccinated was 83.0% overall and ranged from 70.3% in Wyoming to 92.4% in Washington, DC. After Washington, DC, the 5 states with the highest percentage of adults reporting being full vaccinated were New York (90.5%), Vermont (90.6%), Rhode Island (90.9%), Hawaii (91.0%), and Massachusetts (91.5%). By MSA, the percentage of fully vaccinated adults ranged from 77.6% in the Phoenix-Mesa-Chandler, Arizona, MSA to 94.3% in the San Francisco–Oakland-Berkeley, California, MSA (Table 2). In the New York, New York, MSA, the original epicenter of the pandemic, 91.7% of all adults reported being fully vaccinated. Among those who had ever been vaccinated (partially or completely), the reported brand of the first vaccine dose was as follows: Pfizer-BioNtech (52.1%), Moderna (39.2%), Johnson and Johnson (Janssen) (7.0%), one of the brands that requires 2 initial shots, but not sure which brand (0.6%), none of these brands (ie, other, 0.3%), and missing brand information (0.2%). Differences in first administered dose by work setting are shown in the Figure.

**Table 1.** National and State-Specific Prevalence Estimates of Full Vaccination Status and the Proportion Who Had Received a Booster Dose in the Overall Population and Among Those Fully Vaccinated, US, December 1, 2021, to January 10, 2022

Location	Respondents, No.	Population, % (95% CI)		
		Fully vaccinated of total population	Boosted of total population	Boosted of fully vaccinated population
US total	135 821	83.0 (82.6-83.5)	40.2 (39.7-40.8)	48.5 (47.9-49.1)
Alabama	1524	74.4 (70.6-78.1)	29.4 (26.3-32.5)	39.5 (35.6-43.5)
Alaska	1386	76.3 (71.4-81.2)	37.9 (33.9-41.9)	49.6 (45.2-54.1)
Arizona	3579	78.6 (76.1-81.1)	33.6 (31.4-35.8)	42.7 (40.1-45.4)
Arkansas	1639	74.4 (70.3-78.6)	31.5 (28.2-34.7)	42.3 (38.4-46.1)
California	10 272	88.1 (86.2-90.0)	43.3 (41.2-45.4)	49.1 (46.9-51.4)
Colorado	3811	85.7 (83.6-87.7)	46.7 (44.2-49.2)	54.5 (51.8-57.3)
Connecticut	2716	90.0 (88.1-91.9)	50.7 (47.8-53.5)	56.3 (53.4-59.2)
Delaware	1433	88.1 (85.3-90.8)	43.3 (39.3-47.4)	49.2 (44.8-53.6)
District of Columbia	2078	92.4 (89.6-95.2)	54.6 (50.0-59.2)	59.1 (54.4-63.8)
Florida	4491	82.1 (79.7-84.4)	36.4 (33.9-38.8)	44.3 (41.5-47.2)
Georgia	3159	75.2 (72.1-78.2)	33.1 (30.4-35.8)	44.1 (40.7-47.4)
Hawaii	1446	91.0 (88.5-93.5)	45.9 (41.7-50.0)	50.4 (46.0-54.8)
Idaho	2088	73.4 (70.3-76.5)	33.6 (30.5-36.6)	45.7 (42.0-49.5)
Illinois	2892	86.5 (84.2-88.8)	42.7 (39.9-45.5)	49.3 (46.3-52.4)
Indiana	2563	79.1 (76.4-81.9)	37.8 (35.0-40.5)	47.7 (44.6-50.9)
Iowa	2161	78.9 (76.2-81.5)	39.7 (36.8-42.7)	50.4 (46.8-54.0)
Kansas	2527	82.2 (79.8-84.7)	40.8 (38.0-43.6)	49.6 (46.5-52.7)
Kentucky	1776	79.0 (75.9-82.2)	38.8 (35.4-42.2)	49.1 (45.1-53.0)
Louisiana	1451	73.4 (69.7-77.2)	32.5 (29.1-35.8)	44.2 (40.0-48.4)
Maine	1570	85.6 (82.3-88.9)	47.3 (43.3-51.3)	55.2 (50.9-59.6)
Maryland	3279	89.7 (87.8-91.6)	46.0 (43.2-48.7)	51.2 (48.3-54.2)
Massachusetts	4247	91.5 (89.8-93.2)	48.9 (46.4-51.3)	53.4 (50.9-55.9)
Michigan	4089	80.2 (78.1-82.4)	45.0 (42.5-47.4)	56.1 (53.4-58.8)
Minnesota	3112	85.2 (83.1-87.3)	46.8 (44.2-49.4)	54.9 (52.1-57.8)
Mississippi	1038	75.1 (70.6-79.6)	29.4 (25.1-33.7)	39.1 (33.8-44.4)
Missouri	2254	78.9 (75.9-81.9)	39.8 (36.7-42.8)	50.4 (47.0-53.9)
Montana	1421	74.3 (70.6-78.0)	40.6 (36.8-44.5)	54.7 (50.3-59.0)
Nebraska	1663	78.8 (76.0-81.5)	36.6 (33.5-39.7)	46.4 (42.8-50.1)
Nevada	2274	83.2 (80.8-85.6)	35.4 (32.5-38.2)	42.5 (39.3-45.7)
New Hampshire	2591	86.3 (83.8-88.8)	44.6 (41.6-47.6)	51.7 (48.4-55.0)
New Jersey	2767	87.8 (85.7-89.9)	46.0 (43.2-48.8)	52.4 (49.5-55.3)
New Mexico	2257	87.8 (85.1-90.4)	50.5 (46.9-54.0)	57.5 (53.8-61.2)
New York	2672	90.5 (88.7-92.4)	42.6 (39.6-45.5)	47.0 (43.9-50.1)
North Carolina	2303	82.1 (79.0-85.2)	39.9 (36.7-43.1)	48.6 (45.0-52.2)
North Dakota	1111	73.1 (69.4-76.7)	35.3 (31.1-39.4)	48.3 (43.1-53.4)
Ohio	2391	79.1 (76.2-82.0)	38.1 (35.3-40.9)	48.2 (45.1-51.4)
Oklahoma	1892	76.3 (73.3-79.3)	32.5 (29.5-35.4)	42.5 (39.0-46.1)
Oregon	3472	85.6 (83.6-87.6)	42.8 (40.4-45.3)	50.1 (47.3-52.8)
Pennsylvania	3690	81.9 (79.3-84.5)	43.4 (40.7-46.1)	52.9 (50.0-55.9)
Rhode Island	1367	90.9 (88.4-93.3)	48.7 (44.8-52.5)	53.5 (49.5-57.6)
South Carolina	1981	75.4 (72.0-78.9)	37.4 (34.3-40.5)	49.6 (46.1-53.1)
South Dakota	1174	76.1 (71.9-80.3)	40.7 (35.8-45.6)	53.5 (48.2-58.8)
Tennessee	2109	75.4 (72.1-78.7)	35.9 (32.7-39.1)	47.6 (43.9-51.3)
Texas	6722	80.9 (78.7-83.1)	34.5 (32.3-36.7)	42.7 (40.1-45.2)
Utah	3772	83.0 (80.6-85.3)	36.6 (34.2-39.0)	44.1 (41.4-46.8)
Vermont	1629	90.6 (87.8-93.4)	60.3 (56.2-64.4)	66.5 (62.4-70.7)
Virginia	3606	85.8 (83.3-88.3)	42.5 (39.8-45.2)	49.5 (46.6-52.5)
Washington	5460	89.2 (87.6-90.9)	45.4 (43.3-47.6)	50.9 (48.6-53.2)
West Virginia	1273	77.6 (73.4-81.9)	41.1 (36.6-45.7)	53.0 (48.0-58.0)
Wisconsin	2332	80.5 (77.8-83.2)	42.7 (39.8-45.6)	53.0 (49.7-56.4)
Wyoming	1311	70.3 (66.2-74.4)	30.9 (27.5-34.3)	44.0 (39.4-48.5)

**Table 2.** Prevalence Estimates of Full Vaccination Status and the Proportion Who Had Received a Booster Dose in the Overall Population and Among Those Fully Vaccinated, by Selected Metropolitan Statistical Areas, US, December 1, 2021, to January 10, 2022

Metropolitan statistical area	Respondents, No.	Population, % (95% CI)		
		Fully vaccinated of total population	Boosted of total population	Boosted of fully vaccinated population
Atlanta–Sandy Springs–Alpharetta, Georgia	2328	79.1 (76.0-82.2)	36.4 (33.3-39.4)	46.0 (42.4-49.5)
Boston-Cambridge-Newton, Massachusetts; New Hampshire	3738	92.3 (90.6-93.9)	49.9 (47.4-52.5)	54.1 (51.5-56.8)
Chicago-Naperville-Elgin, Illinois; Indiana; Wisconsin	2464	90.6 (88.5-92.6)	45.4 (42.3-48.5)	50.1 (46.8-53.5)
Dallas–Fort Worth–Arlington, Texas	2726	79.5 (76.0-83.0)	34.4 (31.3-37.5)	43.3 (39.7-46.8)
Detroit-Warren-Dearborn, Michigan	2367	79.3 (76.4-82.3)	42.3 (39.3-45.3)	53.3 (49.9-56.8)
Houston–The Woodlands–Sugar Land, Texas	2390	81.7 (78.4-85.0)	35.4 (32.2-38.5)	43.3 (39.7-46.9)
Los Angeles–Long Beach–Anaheim, California	2790	87.5 (83.8-91.1)	42.7 (39.1-46.3)	48.8 (45.1-52.6)
Miami-Fort Lauderdale-Pompano Beach, Florida	2114	85.3 (82.2-88.4)	37.4 (33.6-41.3)	43.9 (39.6-48.2)
New York, New York; Newark–Jersey City, New Jersey; Pennsylvania	3492	91.7 (90.0-93.3)	42.2 (39.4-45.0)	46.0 (43.0-49.0)
Philadelphia, Pennsylvania; Camden, New Jersey; Wilmington, Delaware; Maryland	3420	89.2 (87.2-91.3)	47.5 (44.7-50.2)	53.2 (50.3-56.1)
Phoenix-Mesa-Chandler, Arizona	2542	77.6 (74.5-80.6)	31.4 (28.8-33.9)	40.4 (37.3-43.6)
Riverside–San Bernardino–Ontario, California	2276	83.7 (80.5-86.9)	35.2 (31.9-38.5)	42.0 (38.4-45.7)
San Francisco–Oakland-Berkeley, California	3147	94.3 (92.3-96.4)	55.4 (51.9-58.9)	58.7 (55.2-62.2)
Seattle-Tacoma-Bellevue, Washington	3641	91.7 (90.1-93.4)	47.7 (45.0-50.3)	52.0 (49.2-54.7)
Washington, DC; Arlington-Alexandria, Virginia; Maryland; West Virginia	5520	92.1 (90.4-93.8)	51.3 (48.8-53.9)	55.7 (53.1-58.4)

**Figure.**
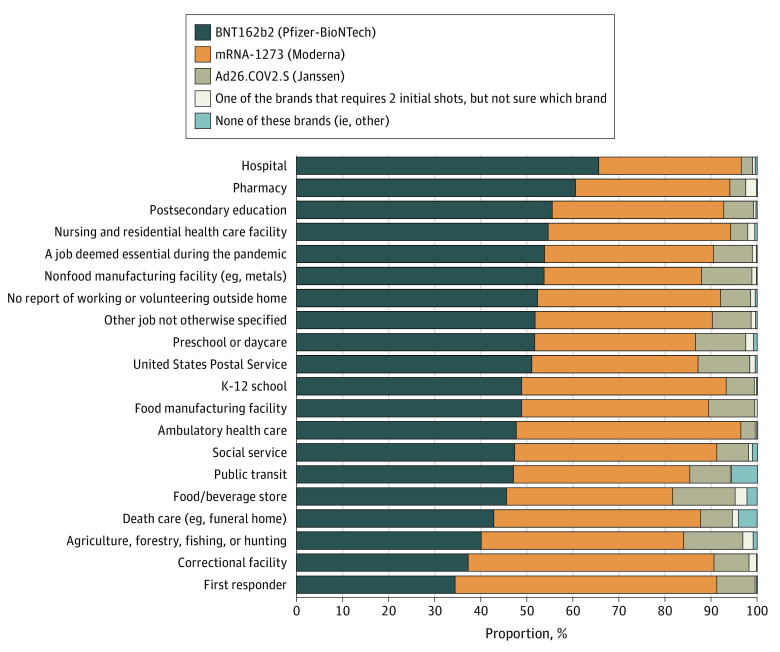
First Administered COVID-19 Vaccine Brand Among Those Who Ever Took the Vaccine, by Occupational Setting, US, December 1, 2021, to January 10, 2022 Occupational setting was defined as the primary location or setting where respondents worked or volunteered outside their home.

### Geographic Variations in Booster Coverage

Of all adults who were fully vaccinated, 48.5% in the US overall had been boosted (Table 1). Expressed as a percentage of the total US adult population, this was 40.2%. By state, booster coverage among the fully vaccinated ranged from 39.1% in Mississippi (or 29.4% of the total adult population), to 66.5% in Vermont (or 60.3% of the total adult population). By MSA, booster coverage among the fully vaccinated ranged from 40.4% in the Phoenix-Mesa-Chandler, Arizona, MSA to 58.7% in the San Francisco–Oakland-Berkeley, California, MSA (Table 2). In the New York, New York, MSA, 46.0% of all fully vaccinated adults had received a booster dose, similar to the 47.0% reported in New York State.

### Sociodemographic Variations in Booster Coverage

Sociodemographic characteristics associated with booster coverage among those fully vaccinated are presented in Table 3 for the US overall. Nationally, the proportion of boosted adults was highest among non-Hispanic Asian individuals (54.1%), those aged 65 years or older (71.4%); those with a doctoral, professional, or master’s degree (68.1%); those who were married with no children in the household (61.2%); those with an annual household income of $200 000 or more (69.3%); those enrolled in Medicare (70.9%); and those working in hospitals (60.5%) or in deathcare facilities such as funeral homes (60.5%). Conversely, only one-third or less of individuals in certain subgroups reported being boosted, including those who ever received a diagnosis of COVID-19; those enrolled in Medicaid; those working in pharmacies (booster coverage was almost 25 percentage points higher among those working in hospitals compared with those working in a pharmacy), correctional facilities, food and beverage stores, or with the US Postal Service; those with less than high school education (34.0%); aged 18 to 24 years; those who were single with a child in the household; or those reporting their financial situation as very difficult. Table 4 presents APRs for factors associated with receiving a booster dose among fully vaccinated adults. Compared with those who did not work outside their home, the likelihood of being boosted was higher among adults working in a hospital (APR, 1.23; 95% CI, 1.17-1.30), ambulatory health care centers (APR, 1.16; 95% CI, 1.09-1.24), and social service (APR, 1.08; 95% CI, 1.01-1.15), whereas lower likelihood was seen among those working in food or beverage stores (APR, 0.85; 95% CI, 0.74-0.96) and those in the agriculture, forestry, fishing, or hunting industries (APR, 0.83; 95% CI, 0.72-0.97). The likelihood of being boosted increased with increasing age but decreased with decreasing educational level. Ever receiving a diagnosis of COVID-19 was associated with a 30% lower probability of receiving a booster dose (APR, 0.70; 95% CI, 0.68-0.73). Similarly, having no health insurance coverage was associated with significantly lower probability of receiving a booster dose compared with having any form of health insurance. All types of health insurance were associated with a higher probability of being boosted compared with being uninsured, including individuals with private insurance (APR, 1.57; 95% CI, 1.41-1.74), Medicare (APR, 1.55; 95% CI, 1.39-1.73), Medicaid (APR, 1.27; 95% CI, 1.13-1.42), VA or Tricare (APR, 1.30; 95% CI, 1.10-1.55), or other type of insurance not otherwise specified (APR, 1.44; 95% CI, 1.20-1.73). Female respondents were less likely than male respondents to receive a booster dose (APR, 0.96; 95% CI, 0.94-0.98), as were those who lived in a mobile house vs a multiunit apartment complex (APR, 0.84; 95% CI, 0.78-0.91) and those residing in the South vs the Northeast (APR, 0.93; 95% CI, 0.91-0.97).

**Table 3.** Prevalence Estimates of Full Vaccination Status and the Proportion Who Had Received a Booster Dose in the Overall Population and Among Those Fully Vaccinated, by Selected Sociodemographic Characteristics, US, December 1, 2021, to January 10, 2022

Characteristic and category	Respondents, No.	Population, % (95% CI)		
		Fully vaccinated of total population	Boosted of total population	Boosted of fully vaccinated population
People living with respondent, No.				
0 (ie, lives alone)	27 585	87.0 (86.2-87.8)	48.4 (47.3-49.5)	55.6 (54.5-56.8)
1	51 809	88.7 (88.1-89.3)	51.9 (51.1-52.7)	58.5 (57.7-59.4)
2	22 365	83.2 (82.2-84.2)	38.0 (36.8-39.2)	45.7 (44.4-47.0)
3	19 230	80.7 (79.4-81.9)	34.0 (32.7-35.3)	42.1 (40.7-43.6)
≥4	14 832	74.6 (73.1-76.0)	26.3 (25.0-27.6)	35.3 (33.6-36.9)
Race and ethnicity				
Hispanic	13 166	82.6 (81.1-84.2)	29.6 (28.0-31.2)	35.8 (34.0-37.7)
Non-Hispanic				
Asian	7136	96.4 (95.5-97.3)	52.2 (49.9-54.4)	54.1 (51.8-56.4)
Black	9943	79.2 (77.5-80.8)	29.2 (27.7-30.8)	36.9 (35.1-38.8)
White	100 620	83.2 (82.6-83.7)	44.7 (44.1-45.3)	53.7 (53.1-54.4)
Other^a^	4956	74.7 (71.5-77.9)	31.3 (28.7-33.9)	41.9 (38.9-44.9)
Gender				
Male	55 250	83.0 (82.2-83.7)	41.1 (40.2-41.9)	49.5 (48.5-50.4)
Female	76 920	83.5 (83.0-84.1)	40.0 (39.3-40.6)	47.8 (47.1-48.6)
Transgender	464	73.0 (59.1-86.9)	25.4 (17.7-33.1)	34.8 (26.5-43.1)
Other gender	1385	70.6 (65.1-76.1)	31.5 (27.1-35.8)	44.6 (39.3-49.9)
Age, y				
18-24	4701	75.9 (73.6-78.2)	18.2 (16.4-20.0)	24.0 (21.7-26.3)
25-44	45 810	76.3 (75.4-77.2)	28.6 (27.8-29.3)	37.4 (36.5-38.4)
45-64	51 019	85.9 (85.2-86.5)	42.1 (41.2-42.9)	49.0 (48.1-49.9)
≥65	34 291	93.1 (92.3-93.9)	66.5 (65.3-67.7)	71.4 (70.2-72.5)
Education				
Less than high school (reference)	2987	72.5 (69.6-75.4)	24.7 (22.0-27.4)	34.0 (30.5-37.5)
High school graduate	15 578	76.3 (75.2-77.5)	31.6 (30.4-32.7)	41.3 (40.0-42.7)
Some college	27 731	82.2 (81.5-83.0)	35.4 (34.5-36.4)	43.1 (42.0-44.2)
Associate degree	13 733	83.0 (81.9-84.0)	37.3 (36.0-38.7)	45.0 (43.5-46.6)
College degree	39 762	91.7 (91.3-92.2)	51.2 (50.4-52.0)	55.8 (54.9-56.6)
Doctoral, professional, or master’s degree	36 030	94.7 (94.3-95.1)	64.4 (63.6-65.2)	68.1 (67.2-68.9)
Household structure				
Married, no children in household	44 155	90.4 (89.8-91.0)	55.3 (54.4-56.2)	61.2 (60.2-62.1)
Married, ≥1 child in household	29 640	79.4 (78.4-80.5)	33.4 (32.4-34.4)	42.1 (40.9-43.3)
Widowed, divorced, or separated, no children in household	23 304	86.9 (85.9-87.8)	47.5 (46.1-48.9)	54.7 (53.2-56.2)
Widowed, divorced, or separated, ≥1 child in household	8141	74.0 (71.6-76.4)	30.0 (28.0-32.0)	40.5 (38.1-42.9)
Single, no children in household	23 028	83.5 (82.2-84.7)	32.7 (31.5-34.0)	39.2 (37.8-40.6)
Single, ≥1 child in household	6197	66.4 (64.0-68.9)	16.4 (14.7-18.1)	24.7 (22.2-27.1)
Unknown marital status	1356	82.7 (76.0-89.3)	40.7 (32.5-48.9)	49.2 (40.0-58.4)
Annual household income, $				
<25 000	13 415	75.0 (73.3-76.7)	27.5 (26.0-28.9)	36.6 (34.8-38.5)
25 000-49 999	22 510	82.6 (81.5-83.6)	35.2 (33.9-36.4)	42.6 (41.2-44.0)
50 000-99 999	34 833	86.3 (85.5-87.1)	44.3 (43.2-45.3)	51.3 (50.2-52.5)
100 000-199 999	29 705	90.3 (89.5-91.1)	53.9 (52.9-55.0)	59.7 (58.6-60.8)
≥200 000	12 221	94.3 (93.4-95.2)	65.4 (63.8-66.9)	69.3 (67.8-70.9)
Unknown income	23 137	76.1 (74.8-77.4)	30.4 (29.3-31.6)	40.0 (38.6-41.5)
Financial difficulty				
Not at all difficult	69 355	89.2 (88.6-89.8)	53.3 (52.5-54.1)	59.7 (58.9-60.6)
A little difficult	27 872	85.4 (84.4-86.3)	37.8 (36.7-38.9)	44.3 (43.0-45.5)
Somewhat difficult	17 641	80.7 (79.5-81.9)	32.3 (31.0-33.7)	40.1 (38.5-41.7)
Very difficult	12 017	70.9 (69.2-72.7)	22.7 (21.4-24.1)	32.0 (30.2-33.8)
Unknown	8936	69.3 (67-71.5)	24.5 (22.8-26.3)	35.4 (33-37.8)
Type of housing				
Mobile housing	4640	70.6 (67.8-73.4)	25.4 (23.1-27.6)	35.9 (32.9-39)
A 1-family house detached from any other house	78 021	85.9 (85.4-86.5)	46.4 (45.7-47.1)	54.0 (53.3-54.8)
A 1-family house attached to ≥1 houses	9375	88.8 (87.2-90.5)	43.6 (41.5-45.7)	49.1 (46.8-51.3)
Multiunit apartment	25 279	84.9 (83.8-86.0)	36.4 (35.1-37.6)	42.9 (41.5-44.2)
Unknown housing	18 506	74.0 (72.5-75.4)	28.0 (26.8-29.3)	37.9 (36.3-39.5)
Employer				
Not working currently	53 093	83.2 (82.4-84.0)	43.8 (42.9-44.7)	52.6 (51.6-53.6)
Government	12 945	88.4 (87.0-89.8)	42.9 (41.1-44.6)	48.5 (46.6-50.4)
Private company	44 338	83.4 (82.6-84.2)	35.7 (34.9-36.6)	42.8 (41.9-43.8)
Nonprofit organization including tax exempt and charitable organizations	10 085	92.8 (91.6-93.9)	54.5 (52.6-56.5)	58.8 (56.8-60.8)
Self-employed	8998	79.0 (77.1-80.9)	38.1 (36.2-40.0)	48.3 (46.0-50.6)
Working in family business	1424	72.7 (66.8-78.6)	30.3 (26.0-34.5)	41.6 (36.4-46.8)
Ever received diagnosis of COVID-19				
No	108 415	86.5 (86.0-87.0)	45.3 (44.7-45.9)	52.4 (51.7-53.0)
Yes	23 999	74.2 (73.0-75.3)	24.1 (23.1-25.1)	32.5 (31.2-33.8)
Not sure	803	59.5 (50.8-68.2)	23.2 (17.1-29.4)	39.0 (30.3-47.8)
Not answered	2604	48.7 (43.2-54.2)	18.2 (14.7-21.6)	37.4 (30.0-44.7)
Health insurance type				
Uninsured	5057	67.2 (64.5-70.0)	16.1 (14.3-17.8)	23.9 (21.4-26.4)
Private	87 182	88.5 (88.1-89.0)	47.1 (46.4-47.8)	53.2 (52.5-53.9)
Medicare, for people aged ≥65 y, or people with certain disabilities	12 031	93.4 (92.4-94.4)	66.2 (64.4-68.0)	70.9 (69.2-72.6)
Medicaid, Medical Assistance, or any kind of government-assistance plan for those with low incomes or a disability	13 943	75.4 (74.0-76.9)	26.5 (25.2-27.9)	35.2 (33.4-36.9)
VA or Tricare	1225	80.0 (73.3-86.6)	30.6 (25.4-35.8)	38.3 (32.1-44.4)
Other insurance	964	71.6 (65.7-77.5)	27.0 (22.3-31.8)	37.8 (31.3-44.2)
Unknown	15 419	72.3 (70.6-74.0)	26.3 (24.9-27.7)	36.4 (34.6-38.2)
Work setting				
No work outside home	82 971	84.4 (83.8-85.1)	42.3 (41.6-43.0)	50.1 (49.3-50.9)
Hospital	3259	96.1 (95.0-97.2)	58.1 (55.1-61.1)	60.5 (57.4-63.6)
Nursing and residential health care facility	1156	88.6 (83.4-93.8)	39.7 (34.5-44.8)	44.8 (39.4-50.2)
Pharmacy	279	91.4 (85.4-97.5)	32.8 (19.3-46.3)	35.9 (20.2-51.6)
Ambulatory health care (eg, doctor, dentist or mental health specialist office, outpatient facility, medical and diagnostic laboratory, home health care)	2518	92.0 (90.0-94.1)	52.4 (48.7-56.1)	56.9 (53.1-60.8)
Social service	2340	88.3 (85.8-90.9)	50.8 (46.9-54.7)	57.5 (53.3-61.7)
Preschool or daycare	540	82.8 (76.7-88.8)	36.1 (29.0-43.3)	43.7 (35.5-51.8)
K-12 school	3975	90.1 (88.5-91.7)	49.6 (46.9-52.2)	55 (52.2-57.8)
Postsecondary education	2335	93.0 (90.7-95.2)	50.0 (46.1-53.9)	53.8 (49.7-57.9)
First responder	817	75.7 (70.1-81.2)	37.4 (31.5-43.4)	49.5 (42.1-56.9)
Deathcare (eg, funeral home)	90	85.4 (75.8-95.0)	51.7 (37.4-65.9)	60.5 (44.9-76.1)
Correctional facility	176	75.2 (62.9-87.5)	26.9 (18.1-35.8)	35.8 (24.4-47.3)
Food or beverage store	1912	81.4 (78.2-84.5)	26.0 (22.7-29.4)	32.0 (27.9-36.1)
Agriculture, forestry, fishing, or hunting	829	66.8 (60.0-73.5)	28.6 (23.0-34.3)	42.9 (35.4-50.4)
Food manufacturing facility (eg, meat-processing)	411	76.4 (69.1-83.6)	28.9 (20.5-37.3)	37.8 (27.2-48.4)
Nonfood manufacturing facility (eg, metals)	1695	73.4 (68.5-78.3)	28.3 (24.7-31.9)	38.5 (34.2-42.9)
Public transit (eg, bus, commuter rail, subway, school bus)	307	83.7 (75.5-91.9)	42.5 (31.1-53.9)	50.8 (38.3-63.3)
US Postal Service	186	76.5 (65.3-87.7)	26.4 (16.7-36.2)	34.6 (22.0-47.1)
Other job deemed essential during the COVID-19 pandemic	8533	78.5 (76.7-80.2)	31.0 (29.2-32.8)	39.5 (37.3-41.7)
Other	15 965	82.6 (81.2-84.1)	39.4 (37.9-41.0)	47.7 (46.0-49.5)
US Census region				
Northeast	23 249	88.0 (87.0-89.0)	45.1 (43.7-46.4)	51.2 (49.8-52.6)
South	41 754	80.0 (79.2-80.9)	36.4 (35.5-37.2)	45.5 (44.4-46.5)
Midwest	28 269	81.2 (80.3-82.1)	41.4 (40.4-42.3)	50.9 (49.8-52.0)
West	42 549	85.9 (84.9-86.9)	42.1 (40.9-43.2)	49.0 (47.7-50.2)

^a^ In the survey, other referred to “Any other race alone, or race in combination.”

**Table 4.** APRs for Factors Associated With Receipt of a COVID-19 Booster Dose Among Those Fully Vaccinated, US, December 1, 2021, to January 10, 2022

Indicator and category	APR (95% CI)	*P* value
Household structure		
Married, no children in household	1 [Reference]	NA
Married, ≥1 child in household	0.89 (0.86-0.92)	<.001
Widowed, divorced, or separated, no children in household	0.96 (0.93-0.99)	.005
Widowed, divorced, or separated, ≥1 child in household	0.82 (0.77-0.87)	<.001
Single, no children in household	0.95 (0.92-0.99)	.03
Single, ≥1 child in household	0.76 (0.69-0.84)	<.001
Unknown marital status	0.89 (0.72-1.11)	.32
Primary location or setting worked or volunteered outside the home in past 7 d		
No work outside home	1 [Reference]	NA
Hospital	1.23 (1.17-1.30)	<.001
Nursing and residential health care facility	1.09 (0.99-1.20)	.07
Pharmacy	0.91 (0.64-1.30)	.61
Ambulatory health care (eg, doctor, dentist or mental health specialist office, outpatient facility, medical and diagnostic laboratory, home health care)	1.16 (1.09-1.24)	<.001
Social service	1.08 (1.01-1.15)	.02
Preschool or daycare	0.98 (0.82-1.18)	.86
K-12 school	1.05 (0.99-1.10)	.08
Postsecondary education	1.04 (0.97-1.11)	.31
First responder	1.09 (0.94-1.27)	.25
Deathcare (eg, funeral home)	1.07 (0.84-1.37)	.57
Correctional facility	0.77 (0.55-1.08)	.13
Food or beverage store	0.85 (0.74-0.96)	.01
Agriculture, forestry, fishing, or hunting	0.83 (0.72-0.97)	.02
Food manufacturing facility (eg, meat-processing)	0.92 (0.70-1.21)	.54
Nonfood manufacturing facility (eg, metals)	0.84 (0.75-0.94)	.002
Public transit (eg, bus, commuter rail, subway, school bus)	1.10 (0.88-1.37)	.39
US Postal Service	0.84 (0.60-1.16)	.28
Other job deemed essential during the COVID-19 pandemic	0.89 (0.85-0.94)	<.001
Other	0.95 (0.91-0.98)	.002
US Census region		
Northeast	1 [Reference]	NA
South	0.93 (0.91-0.97)	<.001
Midwest	0.99 (0.96-1.02)	.52
West	0.99 (0.96-1.03)	.65
Race and ethnicity		
Hispanic	0.92 (0.87-0.96)	<.001
Non-Hispanic		
Asian	1.05 (1.01-1.10)	.02
Black	0.80 (0.76-0.84)	<.001
White	1 [Reference]	NA
Other^a^	0.93 (0.87-1.00)	.046
Gender		
Male	1 [Reference]	NA
Female	0.96 (0.94-0.98)	.001
Transgender	0.96 (0.77-1.19)	.69
Other	1.08 (0.97-1.21)	.15
Age, y		
18-24	1 [Reference]	NA
25-44	1.39 (1.26-1.53)	<.001
45-64	1.78 (1.62-1.97)	<.001
≥65	2.39 (2.16-2.64)	<.001
Highest educational attainment		
Doctoral, professional, or master’s degree	1 [Reference]	NA
College degree	0.90 (0.89-0.92)	<.001
Associate degree	0.75 (0.72-0.77)	<.001
Some college	0.75 (0.73-0.78)	<.001
High school graduate	0.68 (0.66-0.70)	<.001
Less than high school	0.66 (0.60-0.73)	<.001
Type of housing		
Multiunit apartment	1 [Reference]	NA
Mobile housing	0.84 (0.78-0.91)	<.001
A 1-family house detached from any other house	1.06 (1.02-1.09)	.002
A 1-family house attached to ≥1 houses	1.04 (0.99-1.09)	.12
Unknown housing	0.99 (0.92-1.08)	.87
Ever received COVID-19 diagnosis		
No	1 [Reference]	NA
Yes	0.70 (0.68-0.73)	<.001
Not sure	1.00 (0.80-1.26)	.99
Health insurance type		
Uninsured	1 [Reference]	NA
Private	1.57 (1.41-1.74)	<.001
Medicare, for people aged ≥65 y, or people with certain disabilities	1.55 (1.39-1.73)	<.001
Medicaid, Medical Assistance, or any kind of government-assistance plan for those with low incomes or a disability	1.27 (1.13-1.42)	<.001
VA or Tricare	1.30 (1.10-1.55)	.003
Other insurance	1.44 (1.20-1.73)	<.001
Unknown	1.37 (1.20-1.56)	<.001

Abbreviations: APR, adjusted prevalence ratio; NA, not applicable.

^a^ In the survey, other referred to “Any other race alone, or race in combination.”

## Discussion

This survey study’s key findings were that less than half (48.5%) of those who had been fully vaccinated nationwide had received a booster dose, with marked variations seen across geographic locations, occupation types, and other sociodemographic characteristics. Our estimates are similar to national surveillance data from the CDC; of the 189.78 million US adults aged 18 years or older who were fully vaccinated by mid-January 2022, a total of 77.73 million had received a booster dose, yielding a booster coverage rate of 41% among the fully vaccinated.^22^ The difference between the CDC estimate vs ours may reflect the fact that the CDC vaccination surveillance data includes records from territories (Guam, American Samoa, Republic of Palau, Federated States of Micronesia, Northern Mariana Islands, Marshall Islands, and Virgin Islands) and federal entities (eg, Indian Health Service),^22^ whereas our estimate was derived only from the 50 US states and Washington, DC. Moreover, our sample covers only the noninstitutionalized civilian population, whereas the CDC data captures all eligible individuals. In our study, boosted individuals were more likely than nonboosted individuals to be male, Asian, more educated, earning higher income, older, live in the Northeast, and work in hospitals or deathcare facilities. We found wide variations in booster coverage even among workers in the same industry. For example, within health care facilities nationwide, booster coverage was almost 25 percentage points higher among those working in hospitals compared with those working in a pharmacy, even with similar rates of initial vaccination completion within these settings. This wide variability in uptake of booster doses may reflect greater volition in individuals’ decision to receive the vaccine, in contrast to the initial vaccination series for which mandates existed in certain settings.^23^ Mass vaccination campaigns that incentivize people to receive and publicize their booster vaccinations may cue similar action among individuals in similar social networks according to research showing that anecdotal evidence is more influential than statistical evidence in situations where emotional engagement is high (eg, personal or health issues).^24^

Barriers to uptake of booster doses are likely to be different from those against the initial doses. Viewed through the lens of the health belief model components (ie, health motivation, perceived susceptibility, perceived severity, perceived benefits, perceived harms, and cues to action),^25,26,27^ it might be surmised that hesitancy to initial vaccination was largely associated with perceived harm, including fear of unknown adverse effects of COVID-19 vaccines, and/or that some vaccines use the relatively new mRNA platform.^28,29^ On the other hand, the perception of having low susceptibility to COVID-19, or not having a severe illness in the instance of a breakthrough infection, may be factors that may explain relatively low uptake of booster doses. This is suggested by our finding that fully vaccinated individuals who were also COVID-19 survivors had 30% less likelihood of getting a booster dose compared with those with no history of SARS-CoV-2 infection, a finding that might be linked with perceived additional protection from infection-induced immunity.^30^

Uninsured individuals were significantly less likely to receive a booster dose compared with those with insurance. Also, those reporting experiencing very difficult financial situation had half the booster coverage of those reporting no difficulty financially. Booster coverage also decreased with decreasing education and was double among those with a doctoral, professional degree, or master’s degree than those with less than a high school education. Taken together, these findings suggest socioeconomic barriers may limit uptake of the booster doses. Although the vaccine is free within the US, people of low socioeconomic status may also face challenges with scheduling a visit (eg, if they are not internet-enabled), getting time from work, getting transportation, or overcoming language barriers.^31,32,33^ Providing mobile vaccination clinics within low-income neighborhoods on a walk-in basis and launching multilingual awareness campaigns may benefit public health.

### Limitations

This study has limitations. First, data were self-reported and may be subject to social and cognitive biases, which may lead to misreporting. Second, the cross-sectional design of the study means that only associations can be inferred. An important implication of the cross-sectional design is that we are unable to tease apart age, period, and cohort effects. For example, although our results suggested that individuals who ever received a diagnosis of COVID-19 were among the least likely to receive a booster dose, we are unable to assess the timing of diagnosis vs vaccination (eg, whether the diagnosis occurred between the completed vaccination series and the boosting). Thus, we cannot make inferences on whether people got boosted in response to having been infected or got infected despite having been boosted. Third, the results of this study may not be generalizable to individuals outside the sampling frame, including persons in the military, in prisons, or other institutionalized settings. Despite adjustment for differential nonresponse bias, the web-based survey may have resulted in some selection bias to the exclusion of individuals of low socioeconomic status. Fourth, small sample sizes within some subgroups yielded imprecise estimates. Fifth, among the subpopulation of immunocompromised individuals, we may have overestimated the proportion of boosted individuals. According to CDC recommendations, individuals who are immunocompromised should receive a third primary dose.^9^ Because we had no indicator to identify individuals who were immunocompromised, we could not discriminate between a third dose that was a primary dose (among those who were immunocompromised) or a booster dose (among those who were immunocompetent). The magnitude of this bias is, however, likely small as only about 2.7% of the US population is immunosuppressed.^34^

## Conclusions

Of all adults who were fully vaccinated, the percentage who had been boosted was 48.5% in the US overall, with variations seen by geographic, demographic, and occupational characteristics. Boosted individuals are more likely to be male, Asian, more educated, earning higher income, older, live in the Northeast, and work in hospitals. Intensified efforts to increase coverage of booster doses may benefit public health by averting excess premature morbidity and mortality from COVID-19.
